# Survival rate dependent variations in retinopathy of prematurity treatment rates in very low birth weight infants

**DOI:** 10.1038/s41598-020-76472-w

**Published:** 2020-11-10

**Authors:** Jae Hyun Park, Jong Hee Hwang, Yun Sil Chang, Myung Hee Lee, Won Soon Park

**Affiliations:** 1 Department of Pediatrics, Keimyung University Dongsan Hospital, Keimyung University College of Medicine, Daegu, Republic of Korea; 2 Department of Pediatrics, Ilsan Paik Hospital, InJe University College of Medicine, Goyang, Republic of Korea; 3 Department of Pediatrics, Samsung Medical Center, Sungkyunkwan University School of Medicine, 81 Irwon-Ro, Gangnam-gu, Seoul, 06351 Republic of Korea; 4 Statistics and Data Center, Samsung Medical Center, Seoul, Republic of Korea

**Keywords:** Retinopathy of prematurity, Neonatology

## Abstract

As increased oxidative stress causes increased mortality and morbidities like bronchopulmonary dysplasia (BPD) and retinopathy of prematurity (ROP) in very low birth weight infants (VLBWIs), the conundrum of improved survival but increased ROP observed with the high oxygen saturation target range of 91–95% is difficult to explain. To determine the survival rate-dependent variation in ROP treatment rate, 6292 surviving eligible VLBWIs registered in the Korean Neonatal Network were arbitrarily grouped according to the survival rate of infants at 23–24 weeks’ gestation as group I (> 70%, n = 1626), group II (40–70%, n = 2984) and group III (< 40%, n = 1682). Despite significantly higher survival and lower BPD rates in group I than in groups II and III, the ROP treatment rate was higher in group I than in groups II and III. However, the adjusted odds ratios for ROP treatment were not significantly different between the study groups, and the ROP treatment rate in the infants at 23–24 weeks’ gestation was 21-fold higher than the infants at ≥ 27 weeks’ gestation. The controversial association between improved survival and reduced BPD reflecting quality improvement of neonatal intensive care but increased ROP treatment rate might be primarily attributed to the improved survival of the most immature infants.

## Introduction

Increased oxidative stress is a causative factor for increased mortality and morbidities such as bronchopulmonary dysplasia (BPD) and retinopathy of prematurity (ROP) in very low birth weight infants (VLBWIs)^1–7^. Hence, the controversial association between improved survival and increased rates of ROP and BPD with higher oxygen saturation targets of 91–95% that have been observed in several randomized trials and meta-analyses^8–20^ is difficult to explain. Prematurity itself was reported to be a dominant risk factor, with infants born at < 25 weeks’ gestation having 20-fold higher chance of developing severe ROP than those born at 28 weeks’ gestation^11^. Therefore, these findings suggest that the conundrum of association between improved survival reflecting quality improvement of neonatal intensive care and increased ROP treatment rate might be primarily attributed to improved survival of the peri-viable infants born at < 25 weeks’ gestation at the highest risk for developing severe ROP requiring treatment^2,7,21,22^.

Single neonatal intensive care unit (NICU) based studies might have introduced bias into the results due to small sample sizes and/or variation in clinical practices. Therefore, a population based nationwide registry study might be the best way to analyze the complex relationship between mortality and prevalence of ROP and BPD in VLBWIs^23–26^. The Korean Neonatal Network (KNN) is a nationwide, multicenter, prospective and web-based cohort registry system for VLBWIs^27–29^. We observed in our previous studies that the survival rate in peri-viable infants born at 23–24 weeks’ gestation reflected the quality of perinatal and neonatal intensive care of each NICU, and improved survival of infants at 23–24 weeks’ gestation from ≤ 50 to > 50% was associated with significantly less BPD but not severe ROP in the more mature infants at 25–26 weeks’ gestation^28,30,31^. In our previous single center studies, improved survival of infants at 23–24 weeks’ gestation from 55 to 84% was associated with reduced incidence of not only BPD but also ROP in the more mature infants at 25–26 weeks’ gestation^30,32^. In the present study, we further subdivided the KNN data of VLBWIs according to the survival rate of infants at 23–24 weeks’ gestation at < 40%, 40–70% and > 70%, and stratified according to the presence or absence of BPD and gestational age as 23–24, 25–26 and ≥ 27 weeks’ gestation subgroups in order to determine whether the controversial association between improved survival and reduced BPD reflecting quality improvement of neonatal intensive care but increased ROP treatment might be primarily attributed to survival of the most immature infants at the highest risk of severe ROP requiring treatment.

## Results

### Survival and bronchopulmonary dysplasia rate

Table 1 presents the comparison of overall and gestational age-specific survival rates for VLBWIs in the three groups stratified by the survival rates of 23–24 weeks’ gestation. The overall and gestational age-specific survival rate and adjusted odds ratios (ORs) for survival were significantly higher in group I than in groups II and III, and higher in group II than in group III.

**Table 1:** Comparison of overall and gestational age-specific survival rates for very low birth weight infants in the three groups.

Groups	23–24 weeks’ gestation (n = 737)		25–26 weeks’ gestation (n = 1372)		≥ 27 weeks’ gestation (n = 5402)		Total (n = 7511)	
	No. (%)	Adjusted OR (95% CI)^a^	No. (%)	Adjusted OR (95% CI)^a^	No. (%)	Adjusted OR (95% CI)^a^	No. (%)	Adjusted OR (95% CI)^a^
Group I	153/204 (75)^c^	12.42 (3.10, 49.69)	317/358 (89)^c^	3.59 (2.01, 6.41)	1238/1267 (98)^c^	5.15 (3.22, 8.24)	1708/1829 (93)^c^	4.80 (3.45, 6.68)
Group II	185/343 (54)^b^	9.40 (2.50, 35.40)	482/622 (77)^b^	1.10 (0.67, 1.81)	2431/2528 (96)^b^	2.29 (1.65, 3.18)	3098/3493 (89)^b^	2.07 (1.60, 2.68)
Group III	32/190 (17)^b,c^	1 [Reference]	248/392 (63)^b,c^	1 [Reference]	1491/1607 (93)^b,c^	1 [Reference]	1771/2189 (81)^b,c^	1 [Reference]

*OR* odds ratio, *CI* confidence interval.

^a^Adjusted for gestational age, invasive mechanical ventilator (day), non-invasive mechanical ventilator (day) and supplemental oxygen (day).

^b^*p* < .05 compared with group I.

^c^*p* < .05 compared with group II.

Table 2 presents the comparison of overall and gestational age-specific BPD rates for survived VLBWIs in the three groups. The overall and gestational age-specific BPD rate in the infants at 25–26 weeks’ gestation and adjusted ORs for BPD in surviving VLBWIs were significantly lower in group I than in groups II and III, but no significant differences were seen between group II and group III.

**Table 2:** Comparison of overall and gestational age-specific bronchopulmonary dysplasia rates for survived very low birth weight infants in the three groups.

Groups	23–24 weeks’ gestation (n = 367)		25–26 weeks’ gestation (n = 1032)		≥ 27 weeks’ gestation (n = 3904)		Total (n = 4469)	
	No. (%)	Adjusted OR (95% CI)^a^	No. (%)	Adjusted OR (95% CI)^a^	No. (%)	Adjusted OR (95% CI)^a^	No. (%)	Adjusted OR (95% CI)^a^
Group I	104/153 (68)	1.15 (0.14, 9.62)	119/315 (38)^c^	0.50 (0.29, 0.89)	158/1226 (13)^c^	0.81 (0.57, 1.16)	381/1694 (22)^c^	0.68 (0.51, 0.90)
Group II	140/183 (77)	1.10 (0.13, 9.25)	303/481 (63)^b^	0.82 (0.49, 1.37)	501/2419 (21)^b^	0.97 (0.74, 1.28)	944/3083 (31)^b^	0.94 (0.75, 1.19)
Group III	23/31 (74)	1 [Reference]	148/241 (61)^b^	1 [Reference]	337/1468 (23)^b^	1 [Reference]	508/1740 (29)^b^	1 [Reference]

*OR* odds ratio, *CI* confidence interval.

^a^Adjusted for gestational age, invasive mechanical ventilator (day), non-invasive mechanical ventilator (day) and supplemental oxygen (day).

^b^*p* < .05 compared with Group I.

^c^*p* < .05 compared with group II.

### Clinical characteristics

Table 3 demonstrates clinical characteristics according to ROP treatment in the three groups. Clinical characteristics including gestational age and birth weight were significantly lower, and morbidities including BPD and sepsis were significantly higher in surviving VLBWIs with ROP treatment than those without ROP treatment in all three groups. Regardless of ROP treatment, both gestational age and birth weight were significantly lower in group I than in groups II and III, and lower in group II than in group III. BPD and periventricular leukomalacia (PVL) were significantly lower, and symptomatic patent ductus arteriosus (PDA) was significantly higher in group I than in groups II and III.

**Table 3:** Comparison of perinatal characteristics and postnatal morbidities with or without retinopathy of prematurity treatment.

Variables	No ROP treatment (n = 5571)			ROP Treatment (n = 721)			Total (n = 6292)		
	Group I (n = 1412)	Group II (n = 2635)	Group III (n = 1524)	Group I (n = 214)	Group II (n = 349)	Group III (n = 158)	Group I (n = 1626)	Group II (n = 2984)	Group III (n = 1682)
**Perinatal characteristics**									
Gestational age (weeks)	29.1 ± 2.5^b^	29.6 ± 2.5^a^	29.9 ± 2.4^a,b^	25.4 ± 1.7^b^	25.9 ± 1.8^a^	26.6 ± 1.8^a,b^	28.6 ± 2.7^b^	29.1 ± 2.7^a^	29.6 ± 2.5^a,b^
Birth weight (g)	1110.3 ± 255.0^b^	1155.3 ± 233.9^a^	1190.4 ± 218.3^a,b^	755.3 ± 200.8^b^	807.3 ± 220.1^a^	893.0 ± 196.1^a,b^	1063.6 ± 275.9^b^	1114.6 ± 257.8^a^	1162.4 ± 233.0^a,b^
Male, n (%)	703/1412 (50)	1345/2635 (51)	747/1524 (49)	117/214 (55)	163/349 (47)	85/158 (54)	820/1626 (50)	1508/2984 (51)	832/1682 (49)
1-min Apgar score	5.2 ± 1.9^b^	4.9 ± 2.0^a^	5.0 ± 1.7	3.5 ± 1.7	3.4 ± 1.8	3.7 ± 1.8	4.9 ± 2.0^b^	4.7 ± 2.1^a^	4.9 ± 1.8^b^
5-min Apgar score	7.3 ± 1.5^b^	7.1 ± 1.7^a^	7.1 ± 1.4^a^	6.0 ± 1.8	5.7 ± 2.0	6.1 ± 1.7	7.2 ± 1.6^b^	6.9 ± 1.8^a^	7.0 ± 1.5^a^
Small for gestational age, n (%)	347/1412 (25)	660/2635 (25)	393/1524 (26)	37/214 (17)	58/349 (17)	21/158 (13)	384/1626 (24)	718/2984 (24)	414/1682 (25)
Vaginal delivery, n (%)	316/1412 (22)	602/2635 (23)	294/1524 (19)^a,b^	65/214 (30)	89/349 (26)	34/158 (22)	381/1626 (23)	691/2984 (23)	328/1682 (20)^a,b^
Gestational diabetic mellitus, n (%)	113/1412 (8)	216/2635 (8)	133/1524 (9)	9/214 (4)	21/349 (6)	11/158 (7)	122/1626 (8)	237/2984 (8)	144/1682 (9)
Pregnancy-induced hypertension, n (%)	301/1412 (21)	511/2635 (19)	333/1524 (22)	23/214 (11)	37/349 (11)	23/158 (15)	324/1626 (20)	548/2984 (18)	356/1682 (21)
Premature rupture of membrane	371/1409 (26)	612/2618 (23)	361/1507 (24)	76/213 (36)	94/346 (27)	40/153 (26)	447/1622 (28)^b^	706/2964 (24)^a^	401/1660 (24)^a^
Antenatal corticosteroid, n (%)	1203/1394 (86)^b^	1988/2602 (76)^a^	1191/1497 (80)^a,b^	189/212 (89)^b^	267/342 (78)^a^	126/152 (82)	1392/1606 (87)^b^	2255/2944 (77)^a^	1317/1649 (80)^a,b^
Oligohydramnios, n (%)	211/1272 (17)	354/2436 (15)	143/1406 (10)^a,b^	32/200 (16)	43/313 (14)	14/136 (10)	243/1472 (17)	397/2749 (14)	157/1542 (10)^a,b^
Chorioamnionitis, n (%)	477/1332 (36)	759/2258 (34)	291/1067 (27)^a,b^	106/197 (54)	162/307 (53)	39/114 (34)^a,b^	583/1529 (38)	921/2565 (36)	330/1181 (28)^a,b^
**Postnatal morbidities**									
Respiratory distress syndrome, n (%)	1043/1412 (74)	1992/2635 (76)	1178/1524 (77)	213/214 (99)^b^	336/349 (96)^a^	151/158 (96)^a^	1256/1626 (77)	2328/2984 (78)	1329/1682 (79)
Air leak syndrome, n (%)	24/1412 (2)	65/2635 (3)	41/1524 (3)	18/214 (8)	36/349 (10)	4/158 (3)^a,b^	42/1626 (3)	101/2984 (3)	45/1682 (3)
Pulmonary hypertension, n (%)	34/1412 (2)^b^	100/2635 (4)^a^	41/1524 (3)	21/214 (10)^b^	81/349 (23)^a^	19/158 (12)^b^	55/1626 (3)^b^	181/2984 (6)^a^	60/1682 (4)^b^
Symptomatic patent ductus arteriosus, n (%)	446/1352 (33)^b^	658/2626 (25)^a^	390/1517 (26)^a^	129/194 (66)^b^	191/349 (55)^a^	89/158 (56)	575/1546 (37)^b^	849/2975 (29)^a^	479/1675 (29)^a^
Surgical ligation of Patent ductus arteriosus, n (%)	89/1122 (8)^b^	245/2131 (12)^a^	109/1189 (9)^b^	56/175 (32)	131/322 (41)	43/140 (31)	145/1297 (11)^b^	376/2453 (15)^a^	152/1329 (11)^b^
Invasive ventilation (days)	9.4 ± 18.2	9.8 ± 17.9	10.0 ± 22.0	40.0 ± 27.0	48.3 ± 42.0	45.4 ± 46.9	13.4 ± 22.2	14.3 ± 25.4	13.3 ± 27.4
Noninvasive ventilation (days)	16.4 ± 18.4^b^	18.5 ± 20.0^a^	16.7 ± 18.1^b^	36.6 ± 29.5	31.8 ± 23.5	33.9 ± 26.9	19.1 ± 21.4	20.0 ± 20.9	18.3 ± 19.8^b^
Oxygen supplement (days)	6.6 ± 10.9^b^	7.5 ± 12.3^a^	7.8 ± 12.8^a^	14.5 ± 19.3	17.3 ± 20.8	14.5 ± 19.7	7.7 ± 12.6^b^	8.6 ± 13.9^a^	8.4 ± 13.7
Moderate to severe bronchopulmonary dysplasia, n (%)	252/1412 (18)^b^	687/2635 (26)^a^	396/1524 (26)^a^	128/214 (60)^b^	253/349 (72)^a^	107/158 (68)	380/1626 (23)^b^	940/2984 (32)^a^	503/1682 (30)^a^
Sepsis, n (%)	202/1412 (14)	436/2635 (17)	328/1524 (22)^a,b^	80/214 (37)	135/349 (39)	77/158 (49)	282/1626 (17)	571/2984 (19)	405/1682 (24)^a,b^
Necrotizing enterocolitis (Stage ≥ II), n (%)	48/1411 (3)	95/2634 (4)	69/1524 (5)	35/214 (16)	51/349 (15)	15/158 (9)	83/1625 (5)	146/2983 (5)	84/1682 (5)
Intraventricular hemorrhage (Grade ≥ III, n (%)	60/1412 (4)	125/2635 (5)	62/1524 (4)	44/214 (21)	70/348 (20)	27/158 (17)	104/1626 (6)	195/2983 (7)	89/1682 (5)
Periventricular leukomalacia, n (%)	51/1411 (4)^b^	207/2635 (8)^a^	110/1522 (7)^a^	26/213 (12)	55/347 (16)	18/157 (11)	77/1624 (5)^b^	262/2982 (9)^a^	128/1679 (8)^a^
Hospital day	66.2 ± 29.9	65.6 ± 32.0	64.4 ± 28.9	121.2 ± 46.9	121.6 ± 46.4	123.5 ± 50.4	73.4 ± 37.6	72.1 ± 38.4	69.9 ± 35.9^a^

*ROP* retinopathy of prematurity.

^a^*p* < .05 compared with Group I.

^b^*p* < .05 compared with Group II.

### Retinopathy of prematurity treatment rate

Table 4 presents the rate and adjusted ORs of ROP treatment according to the presence or absence of BPD. The ROP treatment rate was significantly higher in surviving VLBWIs with BPD than those without BPD in all three groups. In the comparison between the study groups according to the presence or absence of BPD, the ROP treatment rate was significantly higher in group I than in groups II and III. However, the overall and gestational age-specific adjusted ORs for ROP treatment was not significantly different between the study groups.

**Table 4:** Comparison of retinopathy of prematurity treatment rates with or without bronchopulmonary dysplasia.

Groups	23–24 weeks’ gestation (n = 367)		25–26 weeks’ gestation (n = 1032)		≥ 27 weeks’ gestation (n = 4893)		Total (n = 6292)	
	No. (%)	Adjusted OR^a^ (95% CI)	No. (%)	Adjusted OR^a^ (95% CI)	No. (%)	Adjusted OR^a^ (95% CI)	No. (%)	Adjusted OR^a^ (95% CI)
**Without bronchopulmonary dysplasia**								
Group I	27/49 (55)	1.14 (0.23, 5.53)	38/195 (19)	0.61 (0.33, 1.15)	21/1002 (2)	0.95 (0.51, 1.76)	86/1246 (7)^c^	0.79 (0.52, 1.18)
Group II	21/43 (49)	0.85 (0.17, 4.13)	41/178 (23)	0.82 (0.44, 1.53)	34/1823 (2)	0.86 (0.50, 1.48)	96/2044 (5)^b^	0.80 (0.54, 1.18)
Group III	4/8 (50)	1 [Reference]	24/92 (26)	1 [Reference]	23/1079 (2)	1 [Reference]	51/1179 (4)^b^	1 [Reference]
**With bronchopulmonary dysplasia**								
Group I	70/104 (67)	1.33 (0.48, 3.70)	46/119 (39)	0.89 (0.53, 1.52)	12/157 (8)	0.75 (0.36, 1.57)	128/380 (34)^c^	0.90 (0.63, 1.30)
Group II	101/140 (72)	1.66 (0.61, 4.51)	104/302 (34)	0.89 (0.57, 1.38)	48/498 (10)	1.07 (0.66, 1.76)	253/940 (27)^b^	0.97 (0.71, 1.32)
Group III	15/23 (65)	1 [Reference]	59/146 (40)	1 [Reference]	33/334 (10)	1 [Reference]	107/503 (21)^b,c^	1 [Reference]
**Total**								
Group I	97/153 (63)	1.33 (0.56, 3.13)	84/314 (27)	0.76 (0.51, 1.12)	33/1159 (3)	0.83 (0.52, 1.32)	214/1626 (13)	0.82 (0.63, 1.08)
Group II	122/183 (67)	1.42 (0.61, 3.30)	145/480 (30)	0.90 (0.63, 1.29)	82/2321 (4)	0.97 (0.67, 1.41)	349/2984 (12)	0.91 (0.71, 1.15)
Group III	19/31 (61)	1 [Reference]	83/238 (35)	1 [Reference]	56/1413 (4)	1 [Reference]	158/1682 (9)^b,c^	1 [Reference]

*OR* odds ratio, *CI* confidence interval.

^a^Adjusted for gestational age, invasive mechanical ventilator (day), non-invasive mechanical ventilator (day) and supplemental oxygen (day).

^b^*p* < .05 compared with Group I.

^c^*p* < .05 compared with Group II.

## Discussion

In the present study, we assessed the quality of neonatal intensive care in each hospital solely according to the survival rate of peri-viable infants at 23–24 weeks’ gestation without specific information about care and diagnosis during pregnancy, perinatal and neonatal care, variations in oxygen saturation targets, ROP screening and treatment criteria, and man-power and socioeconomic circumstances. For international comparison, the network ranking according to the survival rate of infants at 24 weeks’ gestation remained largely unchanged as gestational age increased^26^. In our previous and present studies, improved survival rates of infants at 23–24 weeks’ gestation were associated with reduced morbidities such as BPD in the more mature infants at 25–26 weeks’ gestation^28,30,32^. Overall, these findings suggest that quality improvement of perinatal and neonatal intensive care as evidenced by improved survival of peri-viable infants at 23–24 weeks’ gestation could not only improve survival but also reduce morbidity rates of extremely preterm infants^7,28,30,31,33^.

Given that oxygen toxicity increases the risk of death, BPD and ROP in the premature infants^3–6,29,34,35^, the controversial association of decreased ROP, BPD and survival observed in the lower oxygen saturation setting of 85–89%, and the increased ROP, BPD and survival observed in the higher oxygen saturation setting of 91–95%^12–24^ is difficult to explain. For international comparison of neonatal research networks, the Japanese neonatal research network with the highest survival rate and proportion of infants at 24 weeks’ gestation reported the highest BPD and ROP treatment rates, whereas the Swiss neonatal network with a low survival and proportion of infants at 24 weeks’ gestation reported the lowest BPD and ROP treatment rates^2,25^. In the present nationwide population based KNN cohort study of surviving VLBWIs at ≥ 23 weeks’ gestation, despite its best survival and lowest BPD rates indicative of the best quality of neonatal intensive care, the ROP treatment rate was highest in group I compared with group II and/or group III. However, in multivariate analyses, the adjusted ORs for ROP treatment were not significantly different between the study groups. Moreover, the ROP treatment rate was 21-fold higher in the infants at 23–24 weeks’ gestation than in the infants at > 27 weeks’ gestation in this study. Taken together, these findings suggest that as extreme immaturity itself is a primary factor for severe ROP requiring treatment, improved survival of peri-viable infants born at 23–24 weeks’ gestation is primarily responsible for the apparent association between improved survival rate and increased rate of ROP treatment in VLBWIs.

The strengths of this study included the prospective, nationwide, population-based design, which included, at least, one ROP examination on surviving VLBWIs born at ≥ 23 weeks’ gestation during admission. However, the lack of available data on the timing and frequency of the examinations, and the zone and extent of the disease are some limitations of this study.

In conclusion, in this nationwide prospective cohort study of surviving VLBWIs born at ≥ 23 weeks’ gestation with at least, one ROP examination during admission, despite the best survival and lowest BPD rates indicative of the best quality of neonatal intensive care, the highest rate of severe ROP requiring treatment occurred in the group of infants with a survival rate > 70% at 23–24 weeks’ gestation. However, the adjusted odds ratios for ROP treatment were not significantly different between the study groups, and the ROP treatment rate in the infants at 23–24 weeks’ gestation was 21-fold higher than the infants at ≥ 27 weeks’ gestation. The controversial association between improved survival and reduced BPD reflecting quality improvement of neonatal intensive care but increased ROP treatment rate might be primarily attributed to the improved survival of the most immature infants at the highest risk for developing severe ROP requiring treatment.

## Methods

### Patients

**Figure 1:**
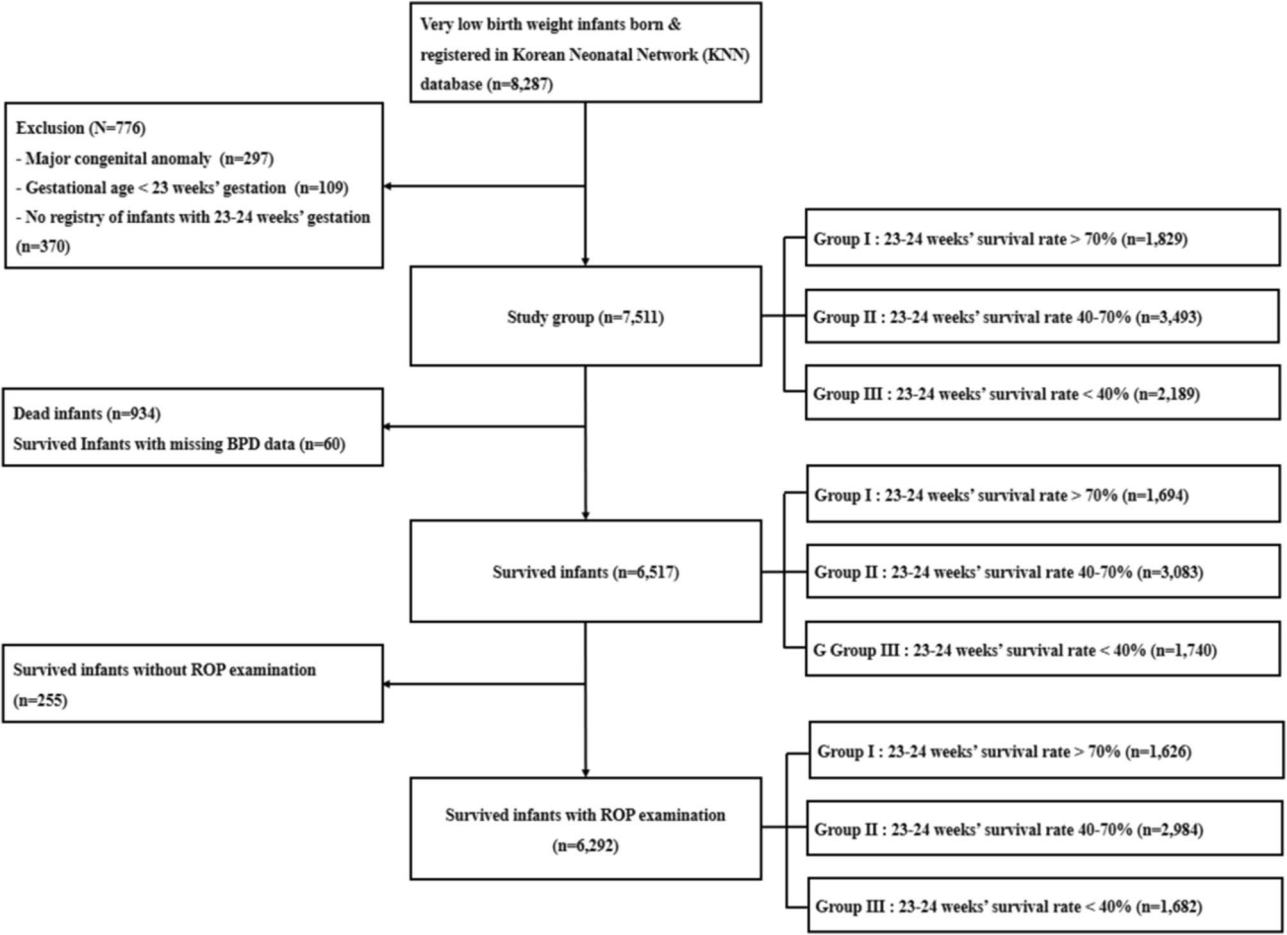
Flowchart showing the study population from the Korean Neonatal Network Database.

The database registry of the KNN prospectively registered the clinical information of VLBWIs defined as birth weight < 1500 g admitted to the 67 voluntarily participating neonatal intensive care units (NICUs) covering > 80% of VLBWIs in South Korea^36^. Between January 1, 2013, and December 31, 2016, 8287 VLBWIs were registered in the KNN database (Fig. 1). As previous studies on the KNN database had already identified prominent institutional differences in the survival rates of infants born at 23–24 weeks’ gestation, we divided the enrolled institutions into three groups based on the survival rate of infants born at 23–24 weeks’ gestation. Of the 67 institutions participating in KNN, only 53 were registered with the KNN database for extremely preterm infants born at 23–24 weeks’ gestation. Therefore, we excluded 370 VLBWIs enrolled at institutions that did not have a registration for infants born at 23–24 weeks’ gestation. To reduce the skew of study outcomes as a result of other causes, we also excluded 297 VLBWIs with major congenital anomalies and 109 born at < 23 weeks’ gestation. Thus, group I included 1829 VLBWIs born at institutions with a survival rate of infants born at 23–24 weeks’ gestation > 70%, group II included 3493 VLBWIs born at institutions with that of 40–70%, and group III included 2189 VLBWIs born at institutions with that < 40%. To investigate the severity of BPD, we excluded 934 infants who died and 60 infants with missing BPD data. To investigate the rate of ROP treatment, we also excluded 225 infants who did not undergo ophthalmic examination before discharge. Considering the difference in survival rate as well as BPD or ROP treatment rates according to gestational age at birth, the infants were stratified into gestational age-specific subgroups of 23–24, 25–26, and ≥ 27 weeks’ gestation.

We recorded the highest stage of ROP using the International Classification of ROP^37^, along with any treatment to prevent vision loss including anti-vascular endothelial growth factor (VEGF) and/or laser ablative and/or surgical treatment^37^, however, other data on the timing and frequency of examination, and zone and extent of disease were not available in this study. We compared perinatal characteristics, including gestational age (GA), birth weight, sex, mode of delivery, Apgar score at 1 and 5 min, presence of small for gestational age (SGA), maternal gestational diabetes mellitus (GDM), pregnancy-induced hypertension (PIH), premature rupture of membrane (PROM), antenatal corticosteroid, oligohydramnios, and chorioamnionitis among the three groups. We also compared neonatal morbidities, including respiratory distress syndrome (RDS), air leak syndrome, pulmonary hypertension, symptomatic PDA, surgical ligation of PDA, BPD, sepsis, necrotizing enterocolitis (NEC), intraventricular hemorrhage (IVH), PVL, and the duration of invasive ventilation representing mechanical respiratory support with invasive artificial airway, non-invasive ventilation representing nasal respiratory support including nasal continuous positive airway pressure or high flow nasal cannula, oxygen supplementation, and hospital stay among the three groups. In addition, we compared the overall and gestational age-specific ROP treatment rates with or without BPD among the three groups.

### Definitions

We complied with the KNN database operation manual to define patient characteristics. In the manual, gestational age was determined from the obstetric history based on the last menstrual period. ROP treatment was defined as any treatment, including anti-VEGF and/or laser ablative and/or surgical treatment, performed on the VLBWIs to prevent visual loss^37^. Maternal steroid use was defined as the administration of any corticosteroid to the mother at any time before delivery to accelerate fetal lung maturity. Chorioamnionitis was confirmed by placental pathology^38^, and PROM was defined as rupture of membranes over 24 h before the onset of labor. BPD was defined as the use of more than supplemental oxygen at 36 weeks’ gestational age, corresponding to moderate to severe BPD using the severity-based definition for BPD of the National Institutes of Health consensus^39^. RDS was defined as respiratory distress requiring ventilator care and surfactant treatment with diagnosis based on chest radiographic findings. Pulmonary hypertension was defined only when accompanied by medical treatment after diagnosis based on echocardiography. Symptomatic PDA was defined as clinical symptoms of PDA, such as ventilator dependence, deteriorating respiratory status, increasing recurrent apnea, pulmonary hemorrhage and hypotension. IVH was defined as grade ≥ 3 according to the classification of Papile et al.^40^ PVL was defined as cystic PVL based on either head ultrasound or cranial magnetic resonance imaging scans performed at ≥ 2 weeks of age^41^. NEC was defined as ≥ stage 2b according to the modified Bell criteria^42^. Sepsis was defined as a positive blood culture in symptomatic infants suggestive of septicemia and more than 5 days of antibiotic treatment^27,28^.

### Statistical analysis

Continuous variables were expressed as mean ± standard deviation (SD) and categorical variables as numbers and proportions. Comparisons between categorical variables were performed using the chi-square test or Fisher’s exact test, and those between continuous variables using one-way analysis of variance (ANOVA). *Post-hoc* tests of one-way ANOVA were used for pairwise comparisons among the three groups, which were arbitrarily divided according to the survival rates of infants born at 23–24 weeks’ gestation. The comparison was further examined in a subgroup analysis with stratification according to gestational ages. A multivariate logistic regression analysis adjusted for covariates such as gestational age, duration of invasive ventilation and non-invasive ventilation, and duration of supplement oxygen was used to estimate the OR with 95% confidence intervals (CI) to identify the relationship between BPD and ROP requiring treatment in the three groups, which were stratified by survival. Analyses were performed using SAS V9.4 (SAS, Cary, NC, USA). A *P* value of < 0.05 was considered statistically significant.

### Ethics statement

The KNN registry was approved by the institutional review board at each participating hospital. Informed consent was obtained from each infant’s parents at enrollment by the NICUs participating in the KNN according to the Korean Privacy Act, and was waived only in the case of infants who died in the delivery room or early after admission to the NICU before informed consent was obtained.
